# Socio – economic determinants of abortion among women in Mozambique and Ghana: evidence from demographic and health survey

**DOI:** 10.1186/s13690-018-0286-0

**Published:** 2018-07-19

**Authors:** Kwamena Sekyi Dickson, Kenneth Setorwu Adde, Bright Opoku Ahinkorah

**Affiliations:** 1 Department of Population and Health, University of Cape Coast, Cape Coast, Ghana; 2 Department of Health, Physical Education and Recreation, University of Cape Coast, Cape Coast, Ghana

**Keywords:** Social – Economic, Abortion, Mozambique, Ghana

## Abstract

**Background:**

Despite the variances in abortion laws accounting for differences in incidence of abortion among African countries, it appears there is absence of literature on other factors that may also account for the differences in incidence of abortion. Specifically, there is paucity of information on how socio-demographic factors account for the disparities in prevalence of pregnancy termination among women of reproductive age in sub-Saharan Africa. In view of this, this paper examined how socio-demographic factors influence pregnancy termination among women in reproductive age in Mozambique and Ghana.

**Methods:**

The study made use of data from the 2014 Ghana and 2011 Mozambique Demographic and Health Survey for the study. For the purpose of this study a sample of 9375 and 13,660 made up of women in their reproductive ages (15–49) in Ghana and Mozambique respectively was used. The results on the analysis of the association between socio-demographic factors and pregnancy termination are presented as odds ratio (OR) with 95% confidence intervals (CI).

**Results:**

The results revealed that about 25% of the respondents in Ghana and 9% of the respondents in Mozambique reported ever had a pregnancy terminated. In both countries, the odds of pregnancy termination were high among women with primary education, those in the older age groups, women who were Christians and women who were employed. Similarly, higher odds of pregnancy termination were found among ever married women, those who less than four births or more and those who have had access to social media (radio and television).

**Conclusion:**

To reduce unintended pregnancies that could lead to pregnancy termination, there is a need for regular integrated community-based outreach programs targeted at generating community responsiveness of effective contraception and prevention of unintended pregnancy.

## Background

Globally, 56 million abortions occur each year, with 22 million being unsafe [1]. According to the World Health Organization (WHO) unsafe abortion as a procedure for terminating a pregnancy performed by persons lacking the necessary skills or in an environment not in conformity with minimal medical standards, or both [2]. Prior to the improvement of medicine, the prevalence of unsafe abortions was high and this exerted a hefty burden on women’s lives. Currently, the introduction of safe and efficient technologies and skills to perform induced abortion is geared in the direction of totally eliminating unsafe abortions and related deaths and providing universal access to these services [3]. In almost all developed countries, there are legal laws that bind safe abortions upon request or under broad social and economic grounds, and abortion services are generally accessible to most women. However, access to safe abortion in some developing countries is limited to a restricted number of narrow conditions [4]. As at 2015, about 90% of women within the childbearing age in Africa lived in countries with restrictive abortion laws [5]. In such countries, a smaller percentage of women satisfy the legal conditions, or are aware of their rights to receive the safe abortion services to which they are legally entitled. Moreover, providers may not be aware of the legal provisions or may be averse to offer legal abortion services [6]. The legal restrictions lead many women to seek services in other countries, from unskilled providers or under unhygienic conditions, exposing them to a significant risk of death or disability [7].

In Africa, countries such as Algeria, Botswana, Gambia, Liberia, Mauritius, Namibia, Sierra Leone and Ghana permit abortion only when it is done with the intention of saving the life of a woman or to preserve her physical and mental health. On the other hand, four countries in Africa (Cape Verde, Mozambique, South Africa and Tunisia) have relatively liberal abortion laws and allow pregnancy termination devoid of restriction as to reason, but with gestational limits [5]. Due the fact that most of abortions are considered illegal and unsafe in Africa, the total prevalence of abortion in Africa was stable between 2003 and 2008, recording almost 29 abortions per 1000 women of childbearing age. The prevalence, however, differed between countries where abortion is legalised and those that have restrictions on abortion [8]. According to the World Health Organization [8], countries in Africa where abortion is legalised have been found to have the lowest abortion rate. On the other hand, countries that have restrictions on abortion usually experience high incidence of abortion. Studies that have been conducted on the practice of abortion in Ghana indicate that at least 47% of women have experienced an abortion at least once in their life [9–12]. An ethnographic research in Mozambique among traditional practitioners indicated abortion continues to be among the most common care sought after [13]. The prevalence of abortion within the Southern Africa subregion, dominated by South Africa was 15 per 1000 women in 2008 [8]. Notwithstanding its rate, abortion remains one of the most neglected global public health challenges. When abortion is considered legal, safe, and is made easily accessible, women’s health improve rapidly. On the contrary, the health of women worsens when there is difficulty in getting access to safe abortion and women resort to unsafe abortions [14].

Studies have also shown that socio-demographic charateristics also affect the utilisation of health care facilities by women in dealing with maternal health issues [15–17]. Nwosu et al. [18] in their study revealed that health seeking behaviour increases with age and deceases at older age. However, Ghosh, Chakrabarti, Chakraborty, et al. [19] also shown in their study that education plays a vital role in the utilisation of health care which in turn influences maternal deaths.

Unsafe abortions account for 70,000 maternal deaths annually and are also responsible for about 5 million women who are suffering from temporary or permanent disability [4, 20]. Globally, developing countries experiece almost all abortion-related deaths, with more deaths occurring in Africa. At least 9% of maternal deaths (16,000) that occur annually in Africa are due to unsafe abortion [5]. Incomplete abortion, excessive loss of blood and infection have been found to be the most common complications that occur as a result of unsafe abortion. Rare, but very serious complications including, septic shock, damage of internal organs and swelling of the peritoneum. Unsafe abortion also has undesirable consequences beyond its immediate effects on women’s health. For example, complications as a results of unsafe abortion can lead to a reduction in women’s productivity, increase the economic burden on poor families, and bring about substantial costs to already struggling public health systems [21]. Despite the differences in abortion laws accounting for differences in prevalence of abortion among African countries, it appears there is inadequate literature on other factors that may also account for the differences in prevalence of abortion. Specifically, there is paucity of information on how socio-demographic factors account for the disparities in prevalence of pregnancy termination among women of reproductive age in sub-Saharan Africa. In view of this, this paper examined how socio-demographic factors influence pregnancy termination among women in reproductive age in Mozambique and Ghana.

## Data and methods

### Data sources

The 2014 Ghana and 2011 Mozambique Demographic and Health Survey data were used for the study. Demographic and Health Survey is a nationwide survey which is designed and conducted every five years. The DHS focuses on child and maternal health and is designed to provide adequate data to monitor the population and health situation in Ghana and Mozambique. Demographic and Health Survey was carried out by the Ghana Statistical Service and Ministerio da Saude - MISAU/Moçambique, Instituto Nacional de Estatística - INE/Moçambique with ICF Macro an international company, giving the technical support needed for the survey through MEASURE DHS. The survey employs a stratified two stage sampling technique. The first stage involves the selection of points or clusters (enumeration areas [EAs]). The second stage is the systematic sampling of households listed in each cluster or EA. All women in their reproductive ages (15–49) belonging to selected households or visitors who slept in the household on the night before the survey were considered for interview. The 2014 version of the Ghana Demographic and Health Survey (GDHS) interviewed 9396 women between the ages 15 and 49 from 12, 831 households covering 427 clusters throughout Ghana. It had a response rate of 97% [22]. Whereas the 2011 version of Mozambique Demographic and Health Survey (MDHS) interviewed 13,745 women between the ages 15 and 49 from 13,718 households throughout Mozambique. It had a response rate of 99.8% [23]. For the purpose of this study a sample of 13,660 was used. Permission to use the data set was given us by the MEASURE DHS following the assessment of a concept note. The dataset is available to the public (www.measuredhs.com).

### Definition of variables

The dependent variable employed for this study was “pregnancy termination” which was derived from the question “have you ever had a terminated pregnancy” and responses were coded 0 = “No” and 1 = “Yes”. Eleven independent variables were used for the study, these were; residence, maternal age, marital status, educational level, wealth status, religion, birth history, and occupation. Others included frequency of watching television, frequency of reading newspapers or magazine and frequency of listening to radio, which were used to as proxy to examine the influence media.

### Measurement of variables

Residence was coded as urban =1 rural = 2, age was categorized into, 15–19 = 1, 20–24 = 2, 25–29 = 3, 20–34 = 4, 35–39 = 5, 40–44 = 6, 45–49 = 7. Marital status was captured as never in union =1, married =2, living with partner =3, widowed =4, divorced =5 and separated = 6. Educational level was classified into four categories: No education = 1, primary = 2, secondary = 3 and higher = 4. Wealth status was categorized in poorest = 1, poorer = 2, middle = 3, richer = 4 and richest = 5. Religion was recoded as Christian =1, Islam =2 and traditional/spiritual/other/no religion = 3. Birth history was also captured as Zero birth =1, one birth = 2, two births = 3, three births = 4 and four births or more = 5. Occupation was also categorized into two thus, unemployed = 1 and employed = 2. Frequency of watching television was captured as “not at all” = 1, “less than once a week” = 2, “at least once a week” = 3. Frequency of reading newspaper or magazine was coded as “not at all” = 1, “less than once a week” = 2, “at least once a week” = 3. Frequency of listening to radio was categorized as “not at all” = 1, “less than once a week” = 2, “at least once a week” = 3.

### Statistical analysis

Pearson Chi – square test was conducted to examine the relationship between background characteristics and pregnancy termination. Next, univariate and multivariate binary logistic analysis were conducted to assess the association between women’s socio-demographic and behaviour factors and pregnancy termination. The results from the logistic regression analysis are presented as odds ratio (OR) with 95% confidence intervals (CI). The binary logistic regression was employed since the dependent variable was a dichotomous variable and it allows the predictions on a mixture of continuous and categorical variables. All the analysis was stratified by country. All analysis was done using the women file from both Ghana and Mozambique separated with the aim of comparison among the countries. All analysis was done using STATA version 13.

## Results

### Background characteristics

Table 1 presents results on respondents who have ever had a pregnancy terminated based on their background characteristics which consist of age, wealth status, level of education, religion, birth history, frequency of listening to radio, frequency of reading newspaper or magazine, frequency of watching television, marital status, residence and occupation. The results revealed that about 25% of the respondents in Ghana and 9% of the respondents in Mozambique reported ever had a pregnancy terminated. Thirty – 4 % and 11% of respondents aged 35–39 years had ever had a pregnancy terminated in Ghana and Mozambique respectively. Again, 27 and 13% of respondents with richest wealth status had ever had a pregnancy terminated in Ghana and Mozambique respectively. In Ghana, 25% of respondents with primary level of education had ever had a pregnancy terminated but in Mozambique, 17% of respondents with higher education had ever terminated a pregnancy (see Table 1).

**Table 1:** Background characteristics and prevalence of pregnancy termination in Ghana (*DHS 2014*) and Mozambique *(DHS 2011)*

Variable	Ghana			Mozambique		
	*N* = 9375	Proportions	X^2^: *P* value	*N* = 13,660	Proportions	X^2^: *P* value
Age			X^2^ = 588: *p* < 0.000			X^2^ = 173: *p* < 0.000
15–19	53	3.3		107	3.5	
20–24	304	18.9		232	9.5	
25–29	443	27.2		241	10.7	
30–34	462	33.8		216	10.9	
35–39	446	34.6		191	11.3	
40–44	322	31.3		108	9.4	
45–49	287	33.5		105	9.6	
Wealth status			X^2^ = 155: *p* < 0.000			X^2^ = 98: *p* < 0.000
Poorest	223	15.4		177	6.8	
Poorer	340	20.8		172	6.8	
Middle	516	26.7		177	6.9	
Richer	603	28.6		259	9.4	
Richest	626	28.6		415	13.0	
Level of education			X^2^ = 6: *p* < 0.126			X^2^ = 31: *p* < 0.000
No education	409	22.9		300	7.0	
Primary	448	27.0		619	9.0	
Secondary	1328	25.0		251	10.8	
Higher	132	22.2		32	17.2	
Religion			X^2^ = 25: *p* < 0.000			X^2^ = 12: *p* < 0.05
Christian	1931	25.7		1121	8.9	
Islam	275	19.5		47	6.4	
Traditional/spiritual/other/no religion	110	24.9		33	11.4	
Birth history			X^2^ = 286: *p* < 0.000			X^2^ = 40: *p* < 0.000
Zero birth	401	13.7		189	6.4	
One birth	376	28.4		222	10.2	
Two births	420	32.0		190	9.7	
Three births	363	32.2		437	8.9	
Four births or more	757	28.2				
Frequency of reading newspaper or magazine			X^2^ = 6: *p* < 0.055			X^2^ = 32: *p* < 0.000
Not at all	1959	25.8		959	8.3	
Less than once a week	208	21.7		114	13.0	
At least once a week	151	24.7		128	10.8	
Frequency of listening to radio			X^2^ = 30:0.000			X^2^ = 21: *p* < 0.000
Not at all	273	18.6		362	7.5	
Less than once a week	768	25.4		292	9.6	
At least once a week	1276	26.1		547	9.4	
Frequency of watching television			X^2^ = 61:0.000			X^2^ = 98: *p* < 0.000
Not at all	426	19.3		631	7.1	
Less than once a week	614	25.4		156	10.4	
At least once a week	1277	26.9		414	12.5	
Marital status			X^2^ = 375:0.000			X^2^ = 146: *p* < 0.000
Never married	378	12.3		107	4.3	
Married	1181	29.9		535	8.8	
Living with partner	430	31.8		355	11.2	
Widowed	74	29.3		45	8.73	
Divorced	83	29.7		29	9.7	
Separated	171	38.3		131	12.2	
Residence			X^2^ = 60:0.000			X^2^ = 83: *p* < 0.000
Urban	1422	28.2		564	11.9	
Rural	895	20.6		637	7.1	
Occupation			X^2^ = 236:0.000			X^2^ = 192: *p* < 0.000
Unemployed	269	12.2		415	5.7	
Employed	2048	28.5		786	12.4	
Terminated pregnancy						
No	7057	74.3		12,459	91.2	
Yes	2317	24.7		1201	8.8	

Source: Computed from Ghana DHS 2014, Mozambique DHS2011

It was also observed that about 26% of respondents in Ghana who are Christians had ever terminated a pregnancy but in Mozambique, 11% of respondents belonging to the Traditional/spiritual/other/no religion had ever terminated a pregnancy. About 32% of respondents in Ghana with three births had ever terminated a pregnancy while in Mozambique, about 10% of respondents with one birth had ever terminated a pregnancy. In Ghana, 26% of respondents who never read newspaper or magazine had ever terminated a pregnancy but in Mozambique, 13% of respondents who read newspaper or magazine less than once a week had ever terminated a pregnancy. In Ghana, 26% of respondents who listened to radio at least once a week had ever terminated a pregnancy while in Mozambique, about 10% of respondents who listened to radio less than once a week had ever terminated a pregnancy (see Table 1).

Results of the study also showed that about 27 and 13% of respondents in Ghana and Mozambique respectively who watched television at least once a week had ever terminated a pregnancy. Again, 38 and 12% of respondents who were separated had ever terminated a pregnancy in Ghana and Mozambique respectively. By residence, 28 and 12% of respondents in Ghana and Mozambique respectively had ever terminated a pregnancy. About 29 and 12% of respondents who were employed in Ghana and Mozambique respectively had ever terminated a pregnancy (see Table 1).

### Binary logistic regression

The binary logistic regression analysis carried out between background characteristics and pregnancy termination can be seen in Table 2. In Ghana, the results showed significant associations between ever terminated a pregnancy and wealth status (poorest), level of education (primary and higher), age, religion, occupation, marital status (married, living together, separated), residence, birth history, frequency of reading newspaper or magazine (at least once a week), frequency of listening to radio, and frequency of watching television (at least once a week). In Mozambique, the results showed significant associations between ever terminated a pregnancy and wealth status (poorest, poorer and middle), level of education (primary and secondary), age, religion (Christian), occupation, marital status, residence, birth history and frequency of watching television (at least once a week).

**Table 2:** Binary logistic regression on pregnancy termination among women in Ghana *(DHS 2014)* and Mozambique (*DHS 2011)*

Independent Variables	Ghana	Mozambique
	OR (95% CI)	OR (95% CI)
Wealth status		
Poorest	0.70* (0.48–1.01)	0.72*(0.51–1.03)
Poorer	0.89 (0.64–1.23)	0.72*(0.52–1.00)
Middle	1.05 (0.83–1.34)	0.73*(0.52–1.00)
Richer	1.04 (0.85–1.26)	0.92(0.73–1.16)
Richest	Ref	Ref
Level of education		
No education	Ref	Ref
Primary	1.35**(1.10–1.67)	1.21**(1.01–1.46)
Secondary	1.13(0.93–1.39)	1.34**(1.02–1.76)
Higher	0.74*(0.53–1.04)	1.14(0.70–1.16)
Age		
15–19	Ref	Ref
20–24	5.61***(3.75–8.41)	2.48***(1.75–3.50)
25–29	8.79***(5.78–13.36)	3.06***(2.13–4.40)
30–34	12.13***(7.81–18.88)	3.37***(2.28–4.97)
35–39	13.62***(8.85–20.96)	3.67***(2.45–5.51)
40–44	11.99***(7.54–19.06)	2.98***(1.95–4.57)
45–49	14.23***(8.94–22.66)	3.22***(2.11–4.89)
Religion		
Christian	1.23*(0.99–1.52)	1.42*(0.96–2.10)
Islam	Ref	Ref
Traditional/spiritual/other/no religion	1.42**(1.03–1.97)	1.50(0.88–2.56)
Occupation		
Unemployed	Ref	Ref
Employed	1.38**(1.13–1.68)	2.05***(1.77–2.38)
Marital status		
Never married	Ref	Ref
Married	1.47**(1.09–2.00)	2.22***(1.58–3.12)
Living together	1.67**(1.24–2.24)	2.32***(1.64–3.28)
Widowed	1.23(0.80–1.88)	1.76**(1.10–2.83)
Divorced	1.11(0.69–1.78)	1.97**(1.09–3.54)
Separated	1.74**(1.18–2.56)	2.38***(1.61–3.50)
Residence		
Urban	Ref	Ref
Rural	0.81*(0.65–1.02)	0.78**(0.61–1.00)
Birth history		
Zero birth	1.71**(1.19–2.46)	2.38***(1.61–3.50)
One birth	1.69***(1.32–2.15)	1.64***(1.64–3.28)
Two births	1.43**(1.15–1.78)	1.76**(1.10–2.83)
Three births	1.23*(1.00–1.52)	2.05***(1.51–2.72)
Four births or more	Ref	Ref
Frequency of reading newspaper or magazine		
Not at all	Ref	Ref
Less than once a week	0.91(0.71–1.16)	1.08(0.83–1.42)
At least once a week	0.68**(0.50–0.92)	0.89(0.68–1.17)
Frequency of listening to radio		
Not at all	Ref	Ref
Less than once a week	1.27**(1.04–1.55)	1.16(0.94–1.44)
At least once a week	1.22*(1.01–1.48)	1.14(0.93–1.39)
Frequency of watching television		
Not at all	Ref	Ref
Less than once a week	1.16(0.96–1.41)	1.07(0.83–1.38)
At least once a week	1.23**(1.04–1.46)	1.24*(0.98–1.57)

*p < 1.0 **p < 0.05 ***p < 0.001 OR = Odds Ratio Ref = reference category

Computed from Ghana DHS 2014, Mozambique DHS2011

The prevalence of ever had a pregnancy terminated was low among women with the poorest wealth status compared to women with richest wealth status in both Ghana and Mozambique, although the association is weaker in Ghana. The OR in Ghana is 0.70 (95% Cl: (0.48–1.01) compared to 0.72 (95% CI: (0.51–1.03) in Mozambique. Women with primary education in both countries were more likely to have ever terminated a pregnancy compared to those with no education, although the association was stronger in Ghana. The OR in Ghana is 1.35 (95% Cl: (1.10–1.67) compared to OR of 1.21 (95% CI: (1.01–1.46) in Mozambique. In Ghana, the likelihood of pregnancy termination was low among women with higher educational level compared to those with no education (OR = 0.74, 95% CI = 0.53–1.04) With age, the prevalence of ever terminated a pregnancy was higher among women 45–49 years compared women 15–19 years, although the association was stronger in Ghana. The OR in Ghana is 14.23 (95% Cl: (8.94–22.66) compared to OR of 3.22 (95% CI: (2.11–4.89) in Mozambique. In both countries, ever had a pregnancy terminated was high among Christians compared to Muslims, although the association is stronger in Mozambique. The OR Ghana is 1.23 (95% CI: (1.99–1.52) in Ghana compared to OR of 1.42 (95% Cl: (0.96–2.10) in Mozambique.

With occupation, the prevalence of ever had a pregnancy terminated was high among employed women in both Ghana and Mozambique compared to unemployed women, although the association is stronger in Mozambique. The OR in Ghana is 1.38 (95% Cl: (1.13–1.68) compared to OR of 2.05 (95% CI: (1.77–2.38) in Mozambique. Women who were separated were more likely to have had a pregnancy terminated compared to women who were never married in both Ghana and Mozambique, although the association was stronger in Mozambique. The OR in Ghana is 1.74 (95% Cl: (1.18–2.56) compared to 2.38 (95% CI: (1.61–3.50) in Mozambique. Women in rural areas were significantly less likely to have ever terminated a pregnancy in both Ghana and Mozambique compared to women in urban areas, although the odds were smaller in Mozambique. The OR in Ghana is 0.81 (95% Cl: (0.65–1.02) compared to 0.78 (95% CI: (0.61–1.00) in Mozambique.

The prevalence of ever had a pregnancy terminated was also high among women with zero birth history in both Ghana and Mozambique, although the association was stronger in Mozambique. The OR in Ghana is 1.71 (95% Cl: (1.19–2.46) compared to 2.38 (95% CI: (1.61–3.50) in Mozambique. Women who read newspaper or magazine in Ghana were less likely to have ever terminated a pregnancy compared to those who never read newspaper or magazine (OR = 0.68, CI = 0.51–0.92). Again, women who listened to radio less than once a week in Ghana were more likely to have ever terminated a pregnancy compared to those who never listened to radio. The prevalence of ever had a pregnancy terminated was high among women who watch television at least once a week compared to those who never watch television, although the association was higher in Mozambique. The OR in Ghana is 1.23 (95% Cl: (1.04–1.46) compared to 1.24 (95% CI: (0.98–1.57) in Mozambique.

## Discussion

The current study observed that the prevalence of ever terminating a pregnancy among women in their reproductive age was high in Ghana (25%) and low in Mozambique (9%). This is consistent with the findings obtained by the World Health Organization [8], where abortion was high among countries that had restrictions on abortion and low in countries where abortion is legalised. In both countries, the study showed a reduction in the odds of terminating a pregnancy among women within the poorest wealth status, women who were unemployed and those from the rural areas. This is consistent with previous studies in China [24], Napal [25] and Ghana [26, 27], where wealth status, employment and residence were found to be associated with prevalence of abortion. The low prevalence of pregnancy termination among women from the rural areas, who are unemployed and with poorest wealth status can be explained by the fact that wealthiest women who mostly live in urban areas and are usually employed are financially empowered and can afford to terminate a pregnancy as compared to poor women [28, 29]. Perhaps these findings explain the increased likelihood of the wealthiest women to have induced abortion. Another possible explanation for the lower odds of pregnancy termination among women in rural areas could be low access to abortion services due to disparities in distribution of health resources among rural and urban areas [30–32].

Our study also found a statistically significant difference in pregnancy termination with level of education in both Ghana and Mozambique. Specifically, women with primary level of education were more likely to have had a pregnancy terminated. Women with higher level of education were also likely to have ever had a pregnancy terminated in Ghana. The relationship between pregnancy termination and level of education is consistent with the findings of previous studies in Ethiopia in which women with higher education were not likely to have induced abortion [33] Studies have also shown that education and employment play a key role in determining whether a woman will keep a pregnancy or terminate it. The observation that women with higher education were less likely to have terminated a pregnancy is consistent with studies that have established a linear relationship between contraceptive use and education [34–36]. Thus, women with higher education are more likely to use contraceptives to avoid unintended pregnancies that are likely to result in abortion. On the contrary, studies in China [24] and Ghana [26, 37] found that more educated women have a higher likelihood of induced abortion.

The likelihood of terminating a pregnancy varied by age with the odds of pregnancy termination high among women 45–49 years. Consistent findings were obtained in previous studies in Ethiopia [33], Ghana [37] and South Africa [38], where abortion was found to be high among older women. On the contrary, other studies in Ghana, Kenya, Nigeria, and Ethiopia [39–42] have found that the prevalence of abortion is high among younger women compared to older ones. In many settings, it is alleged that distribution by age of abortion is high among women in the youngest age groups who want to postpone childbearing and women at the end of their childbearing years, who believe they cannot get pregnant at that age, are most likely to get induced abortions. One elucidation may be the perceived need or lack thereof, for contraception at the end of childbearing years, or lack of awareness or unmet need for contraception at the youngest age groups [37].

It was also observed that Islamic women were less likely to terminate a pregnancy as compared to Christians, traditionalist and women who belonged to other religions. This corroborates the findings of Klutsey [26] and Ahiadeke [39], where prevalence of abortion was found to be high among Christians. The low prevalence of abortion among Muslim women could be attributed to the strict rules and punishments guiding the Islamic religion. Our study also found that women pregnancy termination was low among unmarried women compared to women with other marital status. Consistent with the findings of our study, a study in Nigeria also found high prevalence of abortion among married women compared to never married women [43, 44]. A previous study in South Africa [37] also found that single women were significantly less likely to have a spontaneous abortion or a still birth than to have a live birth. The findings of a study in Nepal [45] explained that the prevalence of abortion was high among unmarried women because never-married women expected to experience more undesirable responses from health care workers, family and friends if they were to have an abortion than were married-women.

With regards to birth history, our study observed that women with no children were more likely to terminate a pregnancy as compared to women with parity 4 and above. One of the possible explanations could be that women with no children are likely to be adolescents, belonging the younger age category. If this is so, then the tendency abortion could be high among women of this age category since the rate of unintended pregnancies that often result in abortion has been found to be high among younger women due to unmet need for family planning [36]. Women who had access to social media (radio and television) were found to be more likely to have had a pregnancy terminated compared to those who had no access to media. The importance of media in providing information about how and where to terminate a pregnancy could account for the association between media exposure and the prevalence of pregnancy termination. Women who have access to social media may also be aware of the abortion laws in their country and are less likely to be stigmatised by society in their quest to have a pregnancy terminated.

## Conclusion

Although similar demographic variables provided an understanding of pregnancy termination among women in reproductive age in Ghana and Mozambique, there were variations in relation to how each demographic variable influenced pregnancy termination. In both countries, the odds of pregnancy termination were high among women with primary education, those in the older age groups, women who were Christians and women who were employed. Similarly, higher odds of pregnancy termination were found among ever married women, those who less than four births or more and those who have had access to social media (radio and television). On the contrary, pregnancy termination was low among women within the poorest wealth status, women from rural areas and women who read newspaper or magazine at least once a week. To reduce unintended pregnancies that could lead to pregnancy termination, there is a need for regular integrated community-based outreach programs targeted at generating community awareness of effective contraception and prevention of unintended pregnancy. Pragmatic steps should be taken to make health systems easily accessible to women seeking abortion services.
